# An Imbalance in the Pro/mature BDNF Ratio Occurs in Multiple Brain Regions During Normal Ageing in Wild-Type Mice

**DOI:** 10.1007/s12031-023-02131-0

**Published:** 2023-06-14

**Authors:** Shaun Cade, Xin-Fu Zhou, Larisa Bobrovskaya

**Affiliations:** Health and Biomedical Innovation, Clinical and Health Sciences, University of South Australia, Adelaide, SA 5000 Australia

**Keywords:** Brain-derived neurotrophic factor, Neurotrophin receptors, Normal brain ageing, p75NTR, Wild type mice

## Abstract

The early transition to Alzheimer’s disease is characterized by a period of accelerated brain atrophy that exceeds normal ageing. Identifying the molecular basis of this atrophy could facilitate the discovery of novel drug targets. The precursor of brain-derived neurotrophic factor, a well characterized neurotrophin, is increased in the hippocampus of aged rodents, while its mature isoform is relatively stable. This imbalance could increase the risk of Alzheimer’s disease by precipitating its pathological hallmarks. However, less is known about how relative levels of these isoforms change in middle-aged mice. In addition, the underlying mechanisms that might cause an imbalance are unknown. The main aim of this study was to determine how precursor brain-derived neurotrophic factor changes relative to its mature isoform with normal brain ageing in wild type mice. A secondary aim was to determine if signaling through the neurotrophin receptor, p75 influences this ratio. An increasing ratio was identified in several brain regions, except the hippocampus, suggesting a neurotrophic imbalance occurs as early as middle age. Some changes in receptors that mediate the isoforms effects were also identified, but these did not correspond with trends in the isoforms. Relative amounts of precursor brain-derived neurotrophic factor were mostly unchanged in mutant p75 mice. The lack of changes suggested that signaling through the receptor had no influence on the ratio.

## Introduction

Alzheimer’s disease (AD) is a common cause of dementia characterized by impairments in cognitive functions (Arvanitakis et al. 2019). The pathological processes of AD may begin years, even decades, before the manifestation of cognitive decline (Schmitz et al. 2016). This long pre-clinical period highlights the need to identify changes in the brain that may predispose people to AD (De Strooper and Karran 2016). The hippocampus is a key brain region involved in learning and memory that atrophies during the pre-clinical stages of AD (Timmers et al. 2019). Intraneuronal tau inclusions, a major pathological hallmark, appear in entorhinal regions then spread into associated areas in a series of stages (Braak and Braak 1995; Timmers et al. 2019). Identifying alterations in middle-age, therefore, could facilitate an understanding of why the hippocampus is susceptible to AD. Dendritic spine density in cornu-ammonis 1 (CA1), the largest subregion of the hippocampus (Adler et al. 2018), is reduced in middle-aged mice (Furukawa et al. 2021). Dendritic spines are actin-rich protrusions along dendrites that serve as sites for excitatory synapses (Boros et al. 2019). Impairments in hippocampal neurogenesis, reduced neuronal activity in the dendate gyrus (DG), and reduced expression of plasticity-related genes have also been found (Bensalem et al. 2016; Furukawa et al. 2021; Weber et al. 2015). These findings suggest that alterations occur in the hippocampus as early as middle age in mice. Further investigation of changes in proteins and receptors that are implicated in synaptic plasticity could strengthen this evidence.

The neurotrophins are growth factors that promote the survival, differentiation, and elongation of developing neurons (Martorana et al. 2018; Rauti et al. 2020). The factors also prevent axonal degeneration in the aged brain, indicating that their neuroprotective functions are retained throughout life (von Bohlen und Halbach et al. 2003). Brain-derived neurotrophic factor (BDNF) is a well characterized neurotrophin that has an important role in the brain. It is required for hippocampal long-term potentiation (LTP), a form of excitatory plasticity that forms long-lasting synapses, and certain types of experience-dependent plasticity (Aarse et al. 2016). BDNF is synthesized as a precursor, proBDNF, which is targeted to the regulated secretory pathway by the sorting receptor Sortilin (Chen et al. 2005). It is known to facilitate long-term depression (LTD), a form of plasticity that shrinks dendritic spines, while impairing LTP at Schaffer-collateral-CA1 synapses (Yang et al. 2014). These synaptic alterations are mediated through its interaction with the neurotrophin receptor p75NTR at some hippocampal synapses, but not all (Garad et al. 2021). By contrast, the mature isoform, mBDNF, strengthens hippocampal synapses following excitatory plasticity (Kellner et al. 2014; Petkova-Tuffy et al. 2021; Rauti et al. 2020). These neurotrophic effects are mediated through its interaction with TrkB and activation of associated down-stream pathways (Niculescu et al. 2018). The opposing effects of proBDNF and mBDNF are a normal part of synaptic plasticity in the hippocampus. proBDNF is necessary for the post-natal development of CA1 dendritic spines in rats and memory consolidation, so its presence alone is not neurodegenerative (Sun et al. 2021). However, an imbalance between these isoforms could disrupt synaptic plasticity and increase susceptibility to neurodegeneration.

Previous studies have analysed BDNF isoforms separately to determine how they are changed in old rodents relative to young (Buhusi et al. 2017; Calabrese et al. 2013; Perovic et al. 2013). These studies show that proBDNF is increased in the aged hippocampus while mBDNF remains stable relative to young adults (Buhusi et al. 2017; Perovic et al. 2013). However, it remains unknown how relative levels of these isoforms are changed in middle-aged mice. In addition, the cause of an imbalance has not previously been investigated so it is unknown why it would occur. The aim of the present study was to determine how the pro/mature BDNF ratio changes in middle-aged wild type (WT) mice relative to young. This ratio was used as the main outcome measure because it reflects how levels of the precursor change relative to the mature isoform. It was hypothesized that a relative increase in proBDNF would occur by middle-age in brain regions such as the hippocampus, pre-frontal cortex and brain stem. Mice were grouped into age categories at close intervals (≤ 3 months) to identify any potential trend lines. An additional analysis was performed using the same data to determine if the pro/mature BDNF ratio more closely correlates to brain age than the BDNF isoforms. Finally, the pro/mature BDNF ratio was measured in adult p75 mutant mice (p75^-/-^) to determine if p75NTR signaling influences BDNF maturation. p75^-/-^ mice express a truncated isoform of the receptor which lacks cysteine-rich repeats in its extracellular domain resulting in impaired neurotrophin binding (von Schack et al. 2001). The mice have increased LTP in the amygdala (Busch et al. 2017), increased dendritic spine density in the dendate gyrus (DG), and improved spatial navigation by adulthood (Dokter et al. 2015). These findings suggest that p75NTR signaling could contribute to some of the hippocampal deficits in middle-aged mice. Consequently, it was hypothesized that impaired p75NTR signaling may restore the pro/mature BDNF ratio.

## Materials & Methods

### Animals

All mice were excess stock from breeding colonies housed in the University Core Animal Facility (University of South Australia). The project did not require approval following a review from the University Animal Ethics Committee. Only a notification to the Animal Ethics Committee was required for the opportunistic collection of tissues from excess stock. Mice were housed in filtered, individually ventilated cages in a room maintained at 20.5–23.5℃ with a 12-hour light/dark cycle. They were fed with autoclaved “Standard Rat and Mouse Chow” (Specialty Feeds, Australia) and supplied with acidified drinking water (pH – 4.0). Male and female WT mice (C57BL/6J) were used to measure age-related changes in BDNF isoforms. The mice were grouped into age categories: 6, 9, 12 and 14 months as close to their chronological age as possible (± 1 month). The p75 mutant mice used in this study express a truncated isoform of p75NTR which lacks cysteine-rich repeats in the extracellular domain (von Schack et al. 2001). The lack of these repeats impairs binding to all neurotrophins (von Schack et al. 2001). Mice homozygous for the exon 3 mutation will be referred to as p75^-/-^ and heterozygotes p75^+/-^.

### Humane Killing, Tissue Preparation & Brain Dissection

All mice were humanely killed according to a standard procedure in the core animal facility of CO_2_ for 5 min. This procedure was approved by the University Animal Ethics Committee. Mice were then transcardially perfused with 10 mL of 1x PBS for 2 min. The perfusion was performed as quickly as possible to minimise protein degradation. The brain was then extracted and placed on tissue paper under a lamp. The brain stem, hippocampus, and pre-frontal cortex were then dissected and collected into tubes. Leftover brain tissue, including the thalamus, hypothalamus, and cortex, but excluding the olfactory bulb and cerebellum, was also collected. Leftover brain tissue will be referred to as remaining brain tissue throughout the text. All tissues were snap-frozen on dry-ice then stored at -80 °C.

### Tissue Homogenization

Remaining brain tissue was pulverized using liquid nitrogen in a mortar and pestle to combine the remaining brain regions into one mixture. Briefly, the mortar, pestle and utensils were pre-cooled on dry ice for several minutes before the brain was placed in the mortar. Liquid nitrogen was then poured into the mortar and the tissue shattered with the pestle and ground into a fine powder. The powder was scooped back into the original tube and kept on dry-ice before being returned to -80℃.

Powdered homogenate was later weighed (n < 30 mg) into screw-cap homogenization tubes (2 mL) containing 2 glass beads. Frozen brain regions (brain stem, hippocampus, and pre-frontal cortex) were weighed directly into tubes without undergoing pulverization. Homogenization tubes and tissue samples were kept on dry ice during weighing then thawed on wet ice before adding RIPA buffer at a 1:20 ratio (mg tissue: µL RIPA buffer). RIPA buffer was prepared by dissolving 1 protease inhibitor tablet, Complete Ultra tablets (ROCHE, Germany, cat#11 836 170 001) into stock buffer (50 mM Tris-Cl pH 7.5, 150 mM NaCl, 1 mM EDTA pH 7.5, 0.5% Triton X, 0.5% Na deoxycholate, 0.1% SDS) at room temperature gently shaking. Brain samples were run through 1 cycle of homogenization (3 × 20 s) using a tissue homogenization machine, Precellys 24 (Bertin instruments, France) at room temperature. Homogenate was transferred over to fresh pre-cooled tubes and centrifuged at 13,600 rpm for 30 min at 4 °C. Supernatant was then transferred to fresh pre-cooled tubes and stored at -80 °C.

### MicroBCA Protein Assay

Homogenised brain tissue was diluted 1:200 with milli-Q water then loaded in duplicate (100 µL/well) onto a 96-well plate. A set of bovine serum albumin (BSA) standards ranging in concentration from 1.25 to 80 µg/mL was included along with a blank control (milli-Q water) for background subtraction. The working reagent was prepared following the manufacturer’s instructions (Thermo Scientific, US, cat #23235). Briefly, reagents A, B and C were diluted in a 25:24:1 ratio respectively to the required volume. The tube was then wrapped in foil and stored in a drawer to protect the solution from light. 100 µL of the freshly prepared solution was added to each well, then the plate gently mixed before being wrapped in foil. The plate was incubated for 2 h at 37 °C. Absorbance was measured at 560 nm using a Victor 3, multi-label plate reader (PerkinElmer, Massachusetts, US). The program “Workout 2.5” was used to measure absorbance with an existing protocol that had previously been configured. Absorbance readings from the BSA standards were used to interpolate concentrations in the samples.

### ELISA

Sandwich ELISA was used to measure mBDNF and proBDNF with capture antibodies previously generated in house (Lim et al. 2015; Zhou et al. 2013). These antibodies, sheep anti-proBDNF polyclonal and mouse anti-mBDNF monoclonal (B34D10), are specific for each respective BDNF isoform (Lim et al. 2015). Capture antibodies were prepared in ice-cold coating buffer (85 mM NaHCO_3_, 140 mM Na_2_CO_3_ in milli-Q water) at 5 µg/mL for α-proBDNF and 1 µg/mL for α-mBDNF. 96-well plates (Thermo Scientific, Maxisorp, nunc-immuno plate, Denmark) were coated with capture antibody (100 µl/well) overnight at room temperature. Wells were then washed with 1x PBS (300 µL/well) X 3 before blocking with 3% BSA (Sigma Aldrich, US, cat #A7906)/1x PBS (160 µL/well) for 1 h at 37℃. mBDNF standards (0-750 pg/mL), proBDNF standards (0–2,000 pg/mL) and brain samples (150–400 µg/mL) were prepared in 1% BSA/1x PBST (1x PBS + 0.1% Tween20) and loaded in duplicate wells. Wells were incubated with standards, samples, and a blank control (1% BSA/1x PBS) (100 µL/well) for 1 h at 37℃. Wells were then washed with 1x PBST (300 µL/well) X 3 before incubation with detection antibody (Sheep, α-mBDNF-S3-biotin; 0.10 µg/mL for proBDNF and 0.09 µg/mL for mBDNF) (100 µL/well) for 1 h at 37℃. Wells were then washed with 1x PBST (300 µL/well) X 3 followed by incubation with Streptavidin-horseradish peroxidase (Str-HRP, 0.1 µg/mL; diluted 1:5,000 with 1% BSA/1x PBST) (100 µL/well) for 1 h at 37℃. Wells were then washed with 1x PBST (300 µL/well) X 3 before incubation with Tetramethylbenzidine (TMB) (Sigma Aldrich, US, cat #T0440) (100 µL/well) in the dark at room temperature for 15 min. The reaction was stopped with 0.1 M HCl (100 µL/well) then absorbance measured at 450 nm (0.1 s) using a Victor 3, multi-label plate reader (PerkinElmer, Massachusetts, US). The program “Wallac 1420” was used to read the plate then data exported into an excel spreadsheet. Concentrations of proBDNF and mBDNF in brain samples were interpolated from the standards.

### Western Blotting

Brain samples were diluted to 1.5 µg/µL with 5x sample buffer (60 mM Tris pH 6.8, 40% glycerol, 2% SDS, 0.1% bromophenol blue, 375 mM DTT) and milli-Q water. Samples were then boiled at 95℃ for 5 min before storage at -20℃. 10% separating gels (10% acrylamide, 25% separating buffer, 0.1% SDS, 7.5% Glycerol in milli-Q water + 25 µL TEMED and 125 µL 10% APS per 30 mL) were cast vertically into double-wide plates (C.B.S. Scientific, US). Stacking gel (10 mL stacking acrylamide, pH 6.8 + 7.5 µL TEMED and 150 µL 10% APS) was then poured on top and brain samples loaded (25 µg of protein) along with a molecular weight marker (BIORAD, AUS, cat#1610374). The separation was run at 120 V for 2 h. The gel was then pre-soaked in used 1x transfer buffer along with sponges and filter paper for 20 min. A 0.45 μm nitrocellulose membrane (Amersham, GE healthcare, cat#10600002) was then cut to size and soaked for several minutes. The sponges, filter paper, gel and membrane were assembled into a cassette then loaded into a transfer apparatus (C.B.S. Scientific, US) with fresh 1x transfer buffer. The transfer was run at 0.1 A overnight.

The following morning, membranes were air-dried for 1 h then washed with Ponceau S (2.5 g Ponceau in 500 mL of 1% acetic acid/milli-Q water) for several minutes followed by reverse osmosis (RO) water. They were then washed with TBST (5 min) X 3, shaking. Membranes were blocked with milk (5% skim milk powder/TBST + 0.05% Na azide) for 1 h at room temperature, shaking. Membranes were incubated with Rabbit anti-PAI-1 (Abcam, cat#ab66705, 1:1,500), Rabbit anti-p75NTR (Home-made, 1:1,000), Rabbit anti-Sortilin (Home-made, 1:10,000), or Goat anti-TrkB (Home-made, 1:3,000) overnight at 4℃ gently shaking. They were then incubated with Mouse anti-β-actin (Home-made, 1:20,000) or Sheep anti-GAPDH (Home-made, 1:5,000) for 1 h at room temperature, shaking. All primary antibodies were prepared in 5% BSA/TBST + 0.05% Na azide, except β-actin (TBST) and GAPDH (5% milk/TBST + 0.05% Na azide). After overnight incubation, membranes were washed with TBST (5 min) X 3, then incubated with Donkey anti-Rabbit (LI-COR, cat#926-68073, 1:20,000), Donkey, anti-Goat (LI-COR, cat#926-32214, 1:20,000), or Goat anti-Mouse (LI-COR, cat#926-32210, 1:20,000) for 1 h at room temperature, shaking. All secondary antibodies were IRDye-conjugated (LI-COR, USA) and diluted in TBST. After incubation, membranes were washed in TBST (5 min) X 3, then milli-Q water (5 min) X 1. Blots were scanned using the Odyssey Clx infrared imaging system (LI-COR, USA) and quantified using Image Studio Lite, version 5.2 (LI-COR, USA). To ensure that comparisons were relative, the signal for each protein target was normalized to the signal for an internal loading control, either GAPDH or β-actin.

### Statistical Analysis

Results from all experiments were analysed using GraphPad Prism version 8.0. Normality tests and Q-Q plots were used to check for a normal distribution. For normal data involving 3 or more groups, one-way ANOVA followed by Tukey multiple comparisons was used. For non-normal data with 3 or more groups, Kruskal-Wallis followed by Dunn’s multiple comparisons was used. For normal data involving 2 groups, an unpaired two-tailed t-test was used. For non-normal data involving 2 groups, the Mann-Whitney test was used. To determine the relationship between BDNF isoforms and age, linear regression was used.

## Results

### No Differences in proBDNF and mBDNF were Detected Between Males and Females

**Fig. 1:**
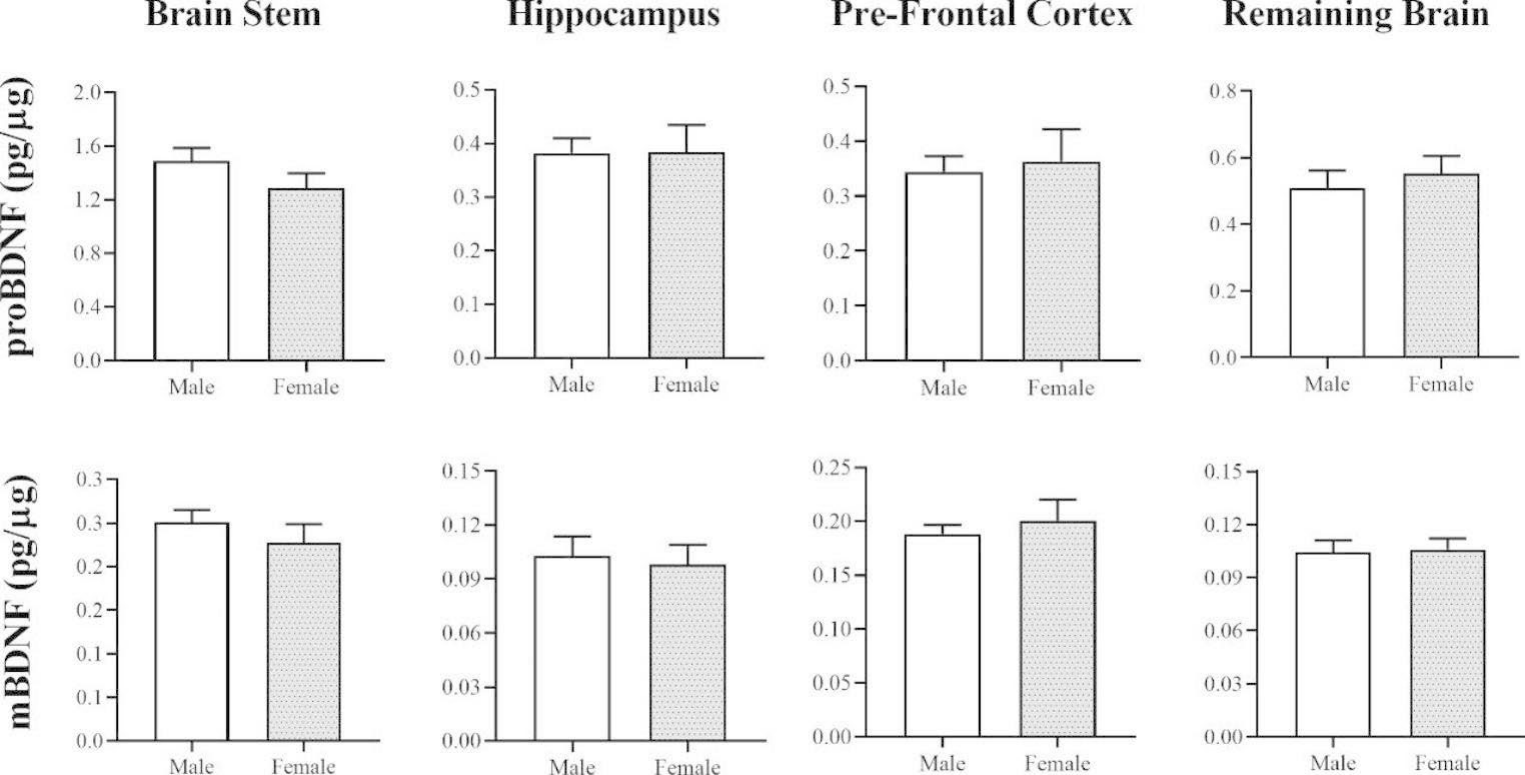
Determining if sex influenced the levels of proBDNF and mBDNF as measured with ELISA in WT mice. Data from each brain region was categorized based on sex for each isoform to detect any differences between males and females. No significant differences were found between males and females for proBDNF in the brain stem (n = 11, males; n = 13, females), the hippocampus (n = 13, males; n = 9, females), the pre-frontal cortex (n = 12, males/females) or the remaining brain tissue (n = 12, males/females) (All unpaired t-tests). Likewise, mBDNF was unchanged between males and females in the brain stem (n = 10, males; n = 13, females) (Unpaired t-test), the hippocampus (n = 13, males; n = 10, females) (Unpaired t-test), the pre-frontal cortex (n = 12, males; n = 11, females) (Mann-Whitney), or the remaining brain tissue (n = 12, males/females) (Mann-Whitney).

To determine if sex influenced levels of BDNF, proBDNF and mBDNF were compared between males and females. Sex-dependent changes in BDNF have previously been identified in mice, suggesting that mixing males and females in the same group could introduce confounding variables (Chan and Ye 2017). As each age category consisted of males and females in this study, it was necessary to confirm that sex did not influence the results. Data obtained from each brain region was categorised based on sex for mice across all the age groups. Unpaired t-tests were then performed to detect any significant differences between males and females. No differences between males and females could be detected in any brain region that was examined for either of the isoforms (Fig. 1). These findings indicate that the inclusion of males and females in each age category did not influence the results.

### proBDNF Increased with age While mBDNF was Mostly Unchanged

**Fig. 2:**
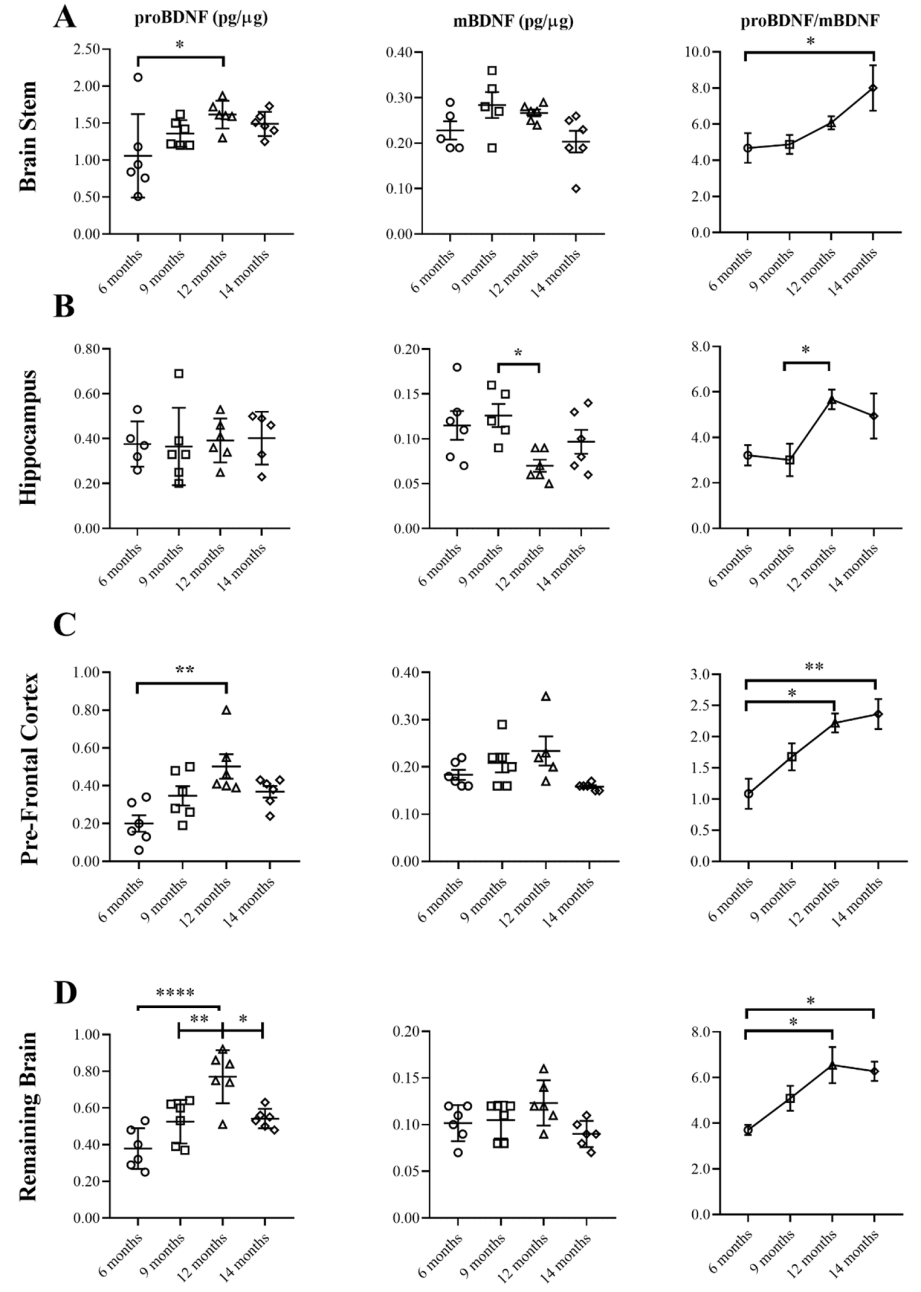
Age-related changes in proBDNF and mBDNF as measured by ELISA in brain regions of WT mice and the pro/mature BDNF ratio. proBDNF was significantly increased in the brain stem of mice aged 12 months relative to 6 months (*p* = 0.0329) (n = 6 per group) however, there were no differences in mBDNF (n = 6: 12, 14 months; n = 5: 6, 9 months) (One-way ANOVA) (**A**). The ratio of pro to mature BDNF also increased in the brain stem of mice aged 14 months relative to 6 months (*p* = 0.0455) (n = 6: 12, 14 months; n = 5: 6, 9 months) (Kruskal-Wallis) (**A**). Although proBDNF remained unchanged in the hippocampus (n = 6: 9, 12 months; n = 5: 6, 14 months), there was a significant reduction in mBDNF in mice aged 12 months relative to 9 months (*p* = 0.0326) (n = 6: 6, 12, 14 months; n = 5: 9 months) (**B**). The ratio of proBDNF to mBDNF showed an increase in the hippocampus of mice aged 12 months compared to 9 months (*p* = 0.0451) (n = 6: 12 months; n = 5: 6, 9, 14 months) (One-way ANOVA) (**B**). Analysis of the pre-frontal cortex showed significantly increased proBDNF in mice aged 12 months relative to 6 months (*p* = 0.0052) (n = 6 per group) (Kruskal-Wallis) (**C**), but no differences in mBDNF (n = 6: 6, 9, 14 months; n = 5: 12 months). The ratio of proBDNF to mBDNF was significantly increased in the pre-frontal cortex of mice aged 12 months compared with 6 months (*p* = 0.0108) and 14 months relative 6 months (*p* = 0.0027) (n = 6: 6, 9, 14 months; n = 5: 12 months) (One-way ANOVA) (**C**). proBDNF was significantly increased in the remaining brain of mice aged 12 months relative to 6 months (*p* < 0.0001), 12 months relative to 9 months (*p* = 0.0057) but reduced in mice aged 14 months relative to 12 months (*p* = 0.0102) (n = 6 per group) (One-way ANOVA) (**D**). No differences in the expression of mBDNF were found for the remaining brain (**D**). The ratio of proBDNF to mBDNF was significantly increased in the brain of mice aged 12 months relative to 6 months (*p* = 0.0290) and 14 months relative to 6 months (*p* = 0.0197) (n = 6 per group) (Kruskal-Wallis) (**D**). Data presented as mean ± SEM, *p < 0.05, **p < 0.01, ***p < 0.001, ****p < 0.0001

proBDNF and mBDNF were measured in brain regions of WT mice to determine how their expression changes with age. proBDNF was increased in all brain regions of mice aged 12 months relative to 6 months except the hippocampus (Fig. 2A and D). For the remaining brain, there was also an increase in mice aged 12 months relative to 9 months (Fig. 2D). However, there were no increases in mice aged 14 months relative to any other age category. Surprisingly, the remaining brain showed a significant reduction in proBDNF at 14 months relative to 12 months (Fig. 2D). By contrast, mBDNF remained unchanged with age for most brain regions except between 9 and 12 months for the hippocampus (Fig. 2B). These results demonstrate that proBDNF increases from 6 to 12 months of age while mBDNF remains relatively unchanged in WT mice.

The pro/mature BDNF ratio was compared across age groups to determine how relative amounts of each isoform change with age. proBDNF was divided by mBDNF so that it could be presented as a multiple of its mature isoform. The ratio increased in WT mice aged 14 months compared to 6 months for all brain regions except the hippocampus (Fig. 2A and D). The ratio was also increased in mice aged 12 months relative to 6 months in the pre-frontal cortex and remaining brain (Fig. 2B and D). Although there was an increase in mice aged 12 months relative to 9 months for the hippocampus (Fig. 2C), the trend line was not the same as for other brain regions.

### The Pro/Mature BDNF Ratio More Closely Reflected Brain Age than Individual Isoforms in Most Brain Regions

**Fig. 3:**
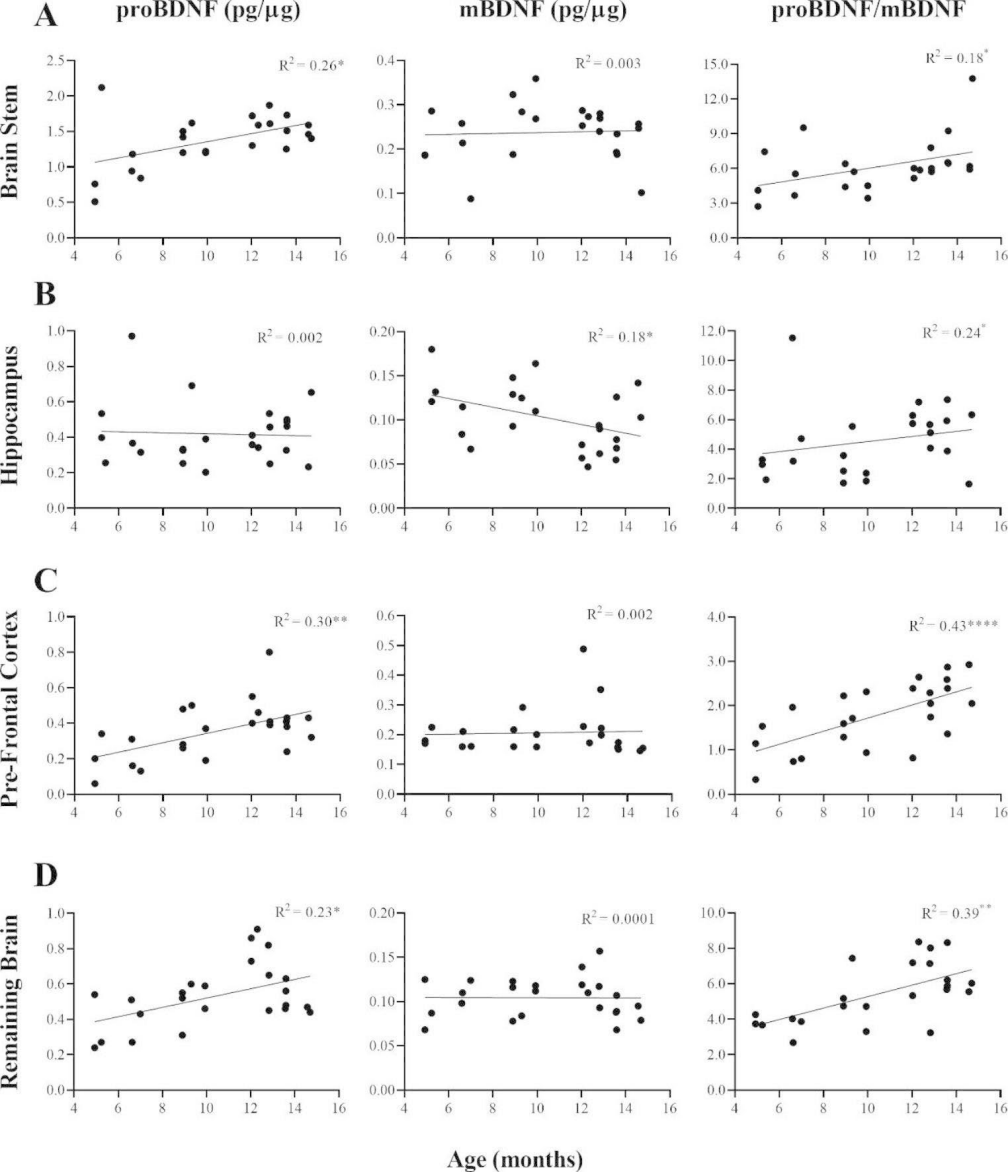
Determining whether the proBDNF/mBDNF ratio correlates more closely with brain age than individual BDNF isoforms. A significant positive correlation between proBDNF and age was found in the brain stem (*p* = 0.0111) (n = 24), but no correlation was found for mBDNF (*p* = 0.8174) (n = 23) (**A**). Although the pro/mature BDNF ratio significantly correlated with age (*p* = 0.0447) (n = 23), the relationship was not as close as for proBDNF (**A**). No correlation between proBDNF and age was identified in the hippocampus (*p* = 0.8184) (n = 24), while mBDNF showed a significant negative correlation (*p* = 0.0376) (n = 24) (**B**). No positive correlation was found between the pro/mature BDNF ratio and age in the hippocampus (*p* = 0.2724) (n = 24) (**B**). For the pre-frontal cortex, proBDNF significantly correlated with age (*p* = 0.0055) (n = 24), while mBDNF showed no correlation (*p* = 0.8290) (n = 24) (**C**). The pro/mature BDNF ratio showed a much closer positive correlation with age than proBDNF (*p* = 0.0006) (n = 24) (**C**). In the remaining brain, a positive correlation for proBDNF was also detected (*p* = 0.0176) (n = 24), but no correlation for mBDNF was found (*p* = 0.9620) (n = 24) (**D**). Consistent with the pre-frontal cortex, the pro/mature BDNF ratio positively correlated more closely with age than proBDNF (*p* = 0.0012) (n = 24) (**D**). Data presented as mean ± SEM, *p < 0.05, **p < 0.01, ***p < 0.001, ****p < 0.0001

Associations between the pro/mature BDNF ratio and age were measured to determine if the ratio is reflective of brain age. The previous analysis indicated that the pro/mature BDNF ratio was more closely related to brain age than either of the BDNF isoforms. To test this hypothesis, correlations between the expression of proBDNF, mBDNF, the pro/mature BDNF ratio and age were compared across brain regions. The precise age of the mice was used in this analysis instead of age categories as previously. The analysis revealed significant positive correlations between the pro/mature BDNF ratio and age in all brain regions (Fig. 3A and D). These correlations were also closer than expression of either BDNF isoform in all brain regions except the brain stem (Fig. 3A). For the brain stem, proBDNF was more closely associated with brain age whereas mBDNF did not show any correlation (Fig. 3A). Only one significant negative correlation was found, which was between the expression of mBDNF and age in the hippocampus (Fig. 3B). The results demonstrated that the pro/mature BDNF ratio more accurately reflects brain age in most brain regions.

### PAI-1 and Sortilin Remained Stable in the Brain Stem While Truncated TrkB Gradually Increased

**Fig. 4:**
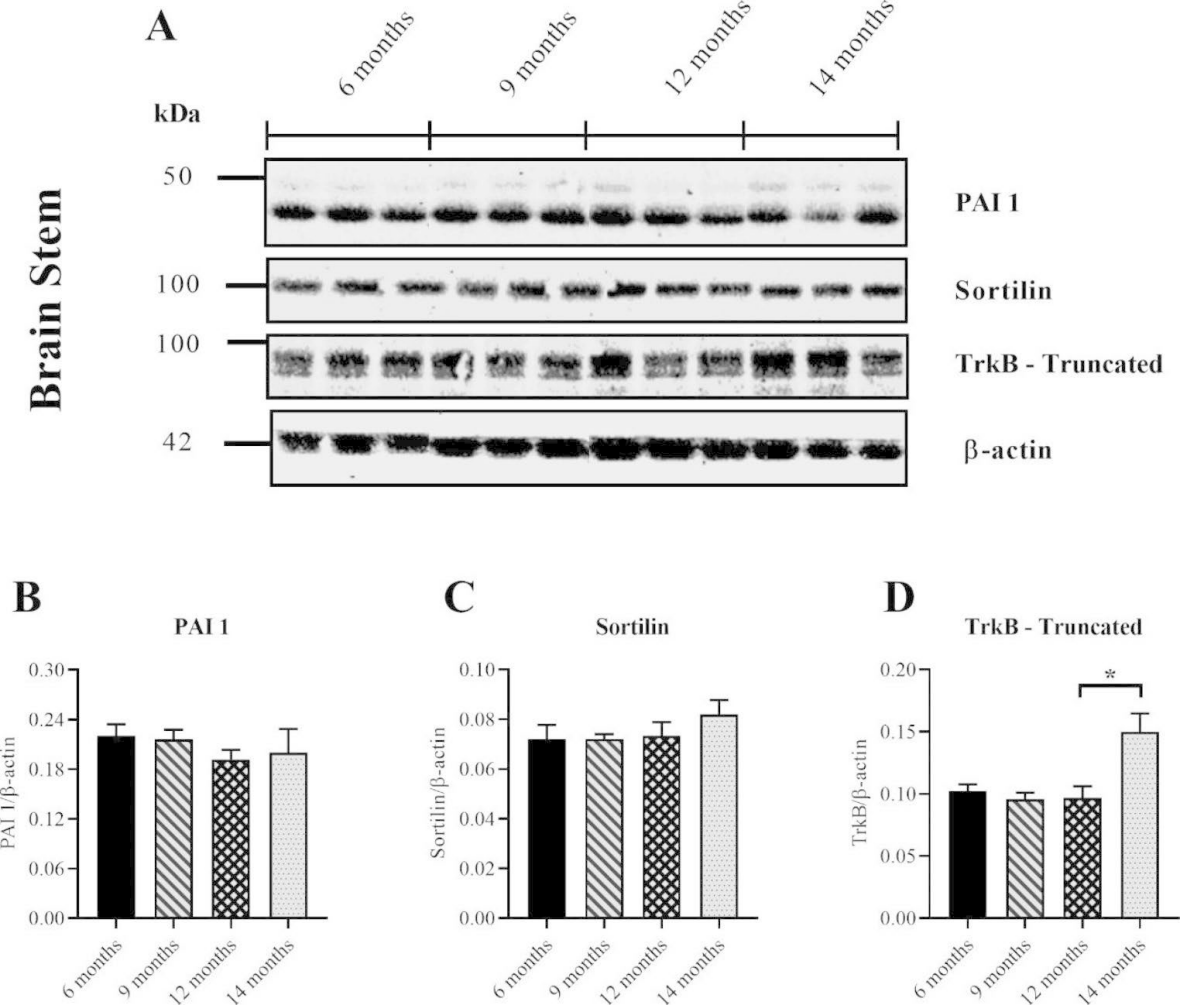
Measuring PAI 1, Sortilin and truncated TrkB in the brain stem of ageing WT mice with western blotting. Representative immunoblots of the tPA inhibitor PAI 1, the vesicle sorting protein Sortilin, the BDNF receptor TrkB and internal loading control β-actin (**A**). Full-length TrkB (~ 145 kDa) could not be detected in the brain stem. No differences could be found for PAI 1 across the groups (*p* = 0.6168) (One-way ANOVA) (**B**). Expression of the vesicle-sorting protein Sortilin also remained unchanged (*p* = 0.4465) (Kruskal-Wallis) (**C**). A significant increase in expression of the truncated isotype of TrkB (~ 95 kDa) was found for mice aged 14 months relative to 12 months (*p* = 0.0213) (Kruskal-Wallis) (**D**). All groups consisted of 5 mouse samples except for the 12 months age group, which consisted of 6 samples. Data presented as mean ± SEM, *p < 0.05, **p < 0.01, ***p < 0.001, ****p < 0.0001

To determine the cause of the increasing pro/mature BDNF ratio in the brain stem, plasminogen-activator inhibitor 1 (PAI-1) was measured. Plasmin is an extracellular protease that converts proBDNF into its mature isoform, mBDNF (Obiang et al. 2011). Plasmin itself is derived from an inactive precursor, plasminogen, by tissue plasminogen activator (tPA), the activity of which is suppressed by PAI-1 (Medcalf 2017). Consequently, an age-related increase in PAI-1 would theoretically reduce tPA activity and, in-turn, the maturation of plasmin, leading to impaired proBDNF conversion. PAI-1 could only be detected in the brain stem, so it was not analysed in any other brain regions. No age-related changes in PAI-1 could be detected in the brain stem (Fig. 4B). This result shows that it remained relatively stable with age.

Sortilin was measured in the brain stem to determine if alterations in the vesicle-associated sorting receptor occur with age. Sortilin targets BDNF to secretory vesicles of the regulated pathway in neurons, suggesting that it plays a key role in its activity-dependent release (Chen et al. 2005). Consequently, age-related increases in Sortilin could potentially increase proBDNF trafficking and disrupt the pro/mature BDNF ratio. No changes in Sortilin were detected in the brain stem with increasing age (Fig. 4C). This result demonstrated that Sortilin remains stable in the brain stem of ageing WT mice.

To determine if BDNF receptors change with age in the brain stem, the TrkB receptor was measured. p75NTR could not be detected in the brain stem, so the analysis was limited to TrkB. 2 isoforms of the TrkB receptor are found in the brain due to alternative splicing of its mRNA transcript (Tessarollo and Yanpallewar 2022). The full-length version (~ 145 kDa) contains a cytoplasmic domain with kinase activity whereas the truncated version (~ 95 kDa), doesn’t (Frisen et al. 1993). Both isoforms have identical extracellular and transmembrane domains which enables them to form homodimers and heterodimers (Tessarollo and Yanpallewar 2022). Because truncated TrkB binds BDNF but lacks intrinsic kinase activity, it is thought that it negatively regulates pro-survival signaling through the full-length isoform (Tessarollo and Yanpallewar 2022). A band was detected at ~ 95 kDa (Fig. 4A) which was thought to correspond to the truncated isoform. Full-length TrkB could not be detected in the brain stem. A significant increase in truncated TrkB was found for mice aged 14 months relative to 12 months (Fig. 4D). This result shows that truncated TrkB increases with age in the brain stem of WT mice.

### Sortilin and Truncated TrkB Remained Stable with Age in the Hippocampus while Full-Length TrkB and p75NTR Steadily Declined

**Fig. 5:**
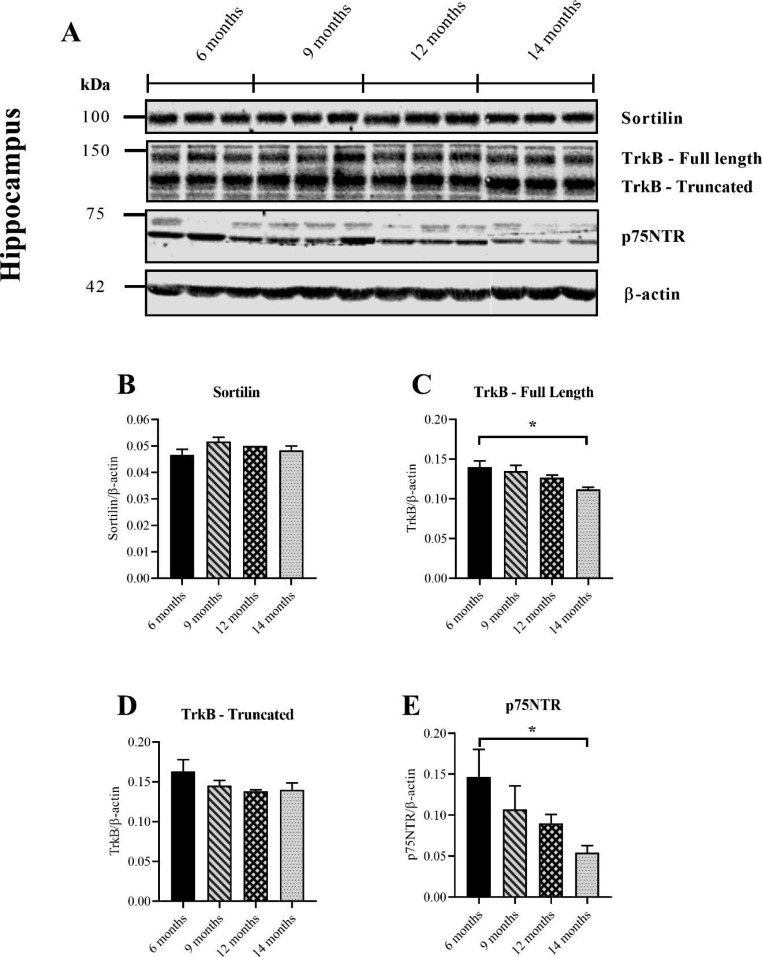
Measuring Sortilin and the BDNF receptor, TrkB in the hippocampus of ageing WT mice with western blotting. Representative immunoblots for the BDNF vesicle sorting protein Sortilin and BDNF receptors (**A**). No differences could be found in expression of Sortilin across the groups (*p* = 0.1733) (Kruskal-Wallis) (**B**). A significant reduction in full-length TrkB (~ 145 kDa) was found for mice aged 14 months relative to 6 months (*p* = 0.0162) (Kruskal-Wallis) (**C**). There were no differences in truncated TrkB across the groups (*p* = 0.5096) (Kruskal-Wallis) (**D**). The expression of p75NTR was significantly reduced in mice aged 14 months relative to 6 months (*p* = 0.0088) (Kruskal-Wallis) (**E**). All age groups consisted of 6 mouse samples except the 14 months age group for truncated TrkB, which consisted of 5 samples. Data presented as mean ± SEM, *p < 0.05, **p < 0.01, ***p < 0.001, ****p < 0.0001

The previous analysis of the pro/mature BDNF ratio in this study revealed that the hippocampus was an exception to other brain regions. No clear trends of an increasing ratio were detected in this brain region for ageing WT mice. Nevertheless, proBDNF and mBDNF receptors were measured across age in the hippocampus to determine if any trendlines emerged. Consistent with the brain stem, the intracellular sorting receptor Sortilin remained stable (Fig. 5B). Together with the brain stem, this result showed that protein levels of Sortilin are not affected by age. Unlike the brain stem, both TrkB isoforms were detected in the hippocampus (Fig. 5A). Each was quantified separately as truncated TrkB may negatively regulate pro-survival signaling through the full-length receptor (Tessarollo and Yanpallewar 2022). Full-length TrkB was reduced in mice aged 14 months relative to 6 months (Fig. 5C). A trend line also appeared showing a gradual decline in the receptor with increasing age (Fig. 5C). By contrast, truncated TrkB did not change with age in the hippocampus (Fig. 5D). The results showed that whilst full-length TrkB declined with age, the truncated isoform did not change. The neurotrophin receptor, p75NTR, was measured to determine how the proBDNF receptor changes with age. proBDNF mediates apoptosis in developing hippocampal neurons and the retraction of neurites through its interaction with p75NTR (Marchetti et al. 2019; Sun et al. 2012). Therefore, a concomitant age-related increase in proBDNF relative to mBDNF and p75NTR would suggest an increase in neurodegenerative signaling. In contrast to the brain stem, p75NTR was readily detectable in the hippocmapus as a band below 75 kDa (Fig. 5A). p75NTR was significantly reduced in WT mice aged 14 months relative to 6 months (Fig. 5E). These results demonstrate that the neurotrophin receptor declined with age in the hippocmapus.

### Age-related Changes in Sortilin and Full-Length TrkB were Detected in the Pre-Frontal Cortex

**Fig. 6:**
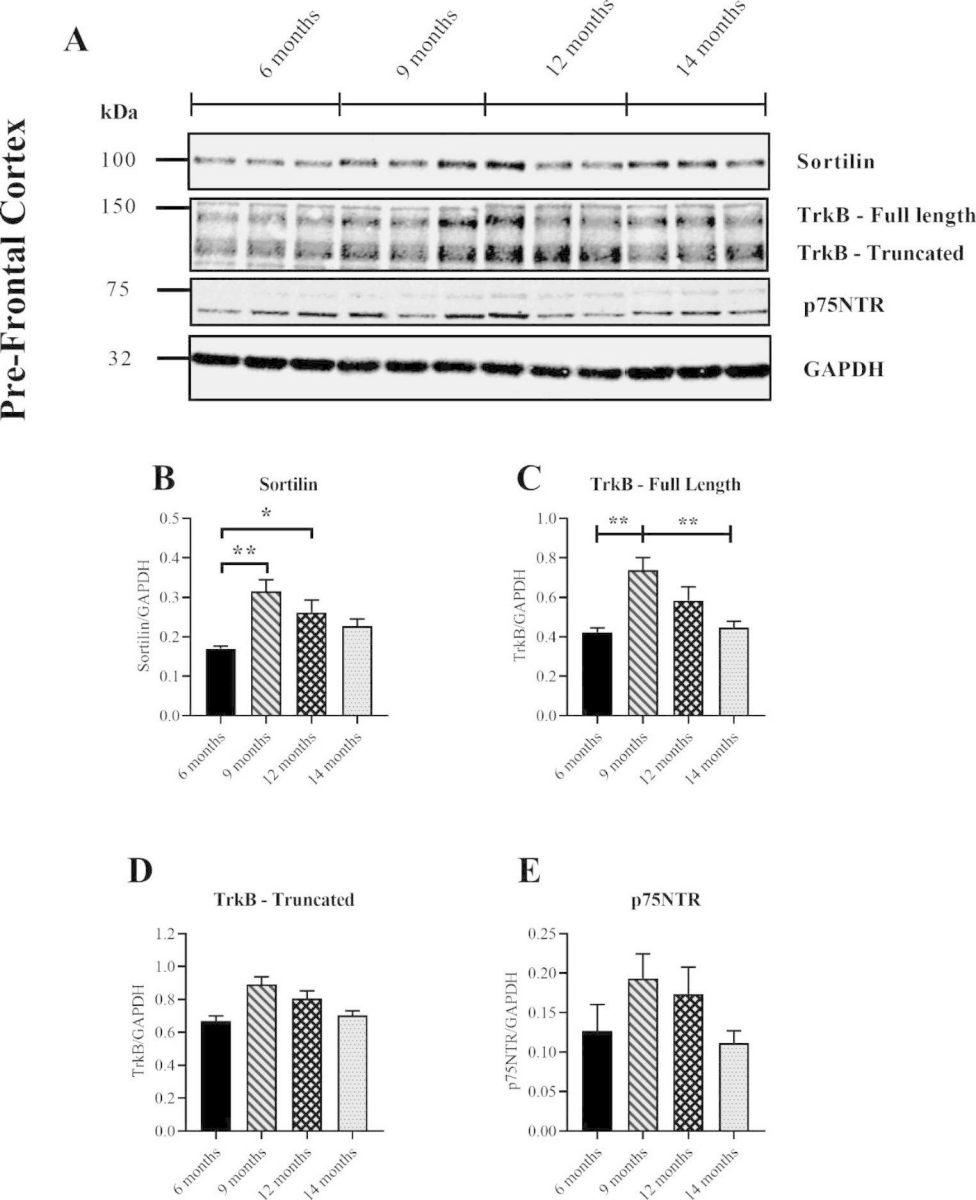
Measuring Sortilin and BDNF receptors in the pre-frontal cortex of ageing WT mice with western blotting. Representative immunoblots of the BDNF sorting receptor Sortilin, the high-affinity receptor TrkB and low-affinity receptor p75NTR (**A**). There were significant increases in Sortilin for both mice aged 9 months relative to 6 months (*p* = 0.0017) and 12 months relative to 6 months (*p* = 0.0494) (Kruskal-Wallis) (**B**). A significant increase in full-length TrkB was found for mice aged 9 months relative to 6 months (*p* = 0.0030) while a decrease was found for mice aged 14 months relative to 9 months (*p* = 0.0039) (One-way ANOVA) (**C**). Truncated TrkB increased in mice aged 9 months relative to 6 months (*p* = 0.0053) and decreased in mice aged 14 months relative to 9 months (*p* = 0.0133) (One-way ANOVA) (**D**) or p75NTR (**E**). Each age group consisted of 6 mouse samples except for the 6 months age group for TrkB, which consisted of 5 samples. Data presented as mean ± SEM, *p < 0.05, **p < 0.01, ***p < 0.001, ****p < 0.0001

The previous analysis in this study revealed that the pro/mature BDNF ratio related to brain age in the pre-frontal cortex more closely than other brain regions. This close association suggested that trendlines in proBDNF and mBDNF receptors may be more likely to emerge in this brain region. A significant increase in Sortilin was detected in mice aged 9 and 12 months relative to 6 months (Fig. 6B). Although there was a decline from 9 months onwards, no significant reductions were detected for mice greater than this age. Full-length TrkB was also significantly increased at 9 months of age relative to 6 months (Fig. 6C). However, the receptor declined from 9 months onwards and was significantly reduced by 14 months (Fig. 6C). By contrast, truncated TrkB remained unchanged with age (Fig. 6D). Like the hippocampus, p75NTR was readily detectable in the pre-frontal cortex as a band below 75 kDa (Fig. 6A). The subsequent quantification did not reveal any significant differences with age despite the apparent changes between groups (Fig. 6E). Moreover, no clear trend line emerged in the pre-frontal cortex, though expression of the receptor appeared to decline from 9 months onwards.

### Sortilin, Full-length TrkB and p75NTR were Unchanged with Age in the Remaining Brain, but Truncated TrkB Steadily Increased

**Fig. 7:**
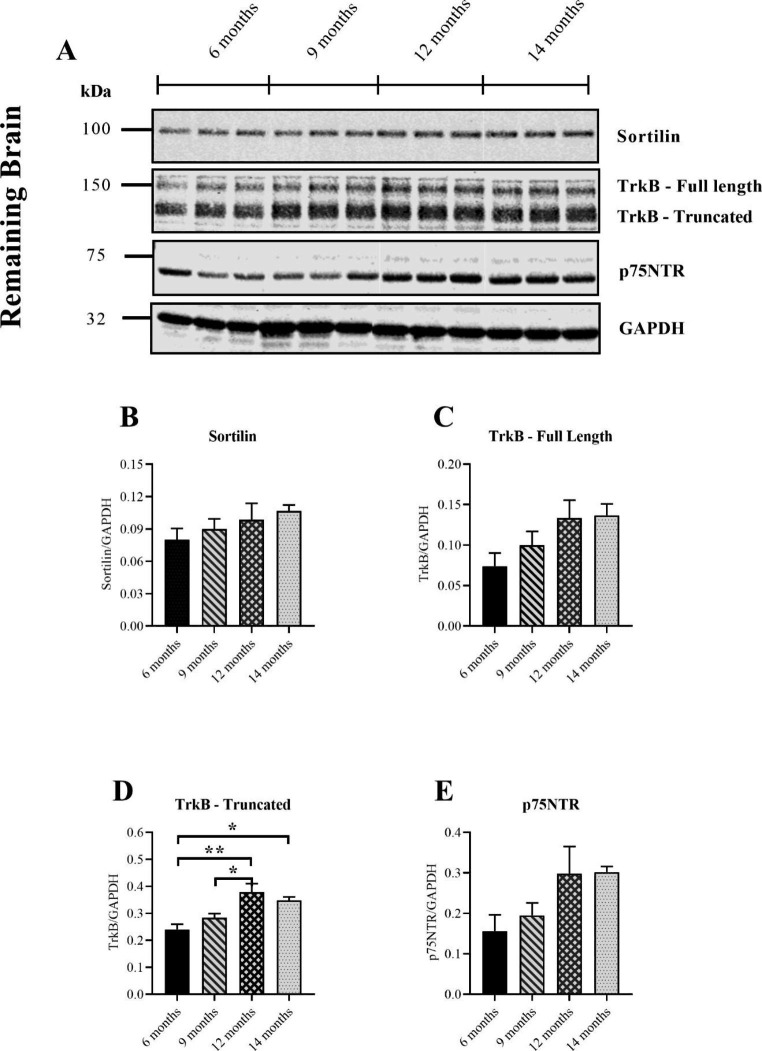
Measuring expression of Sortilin and BDNF receptors in the remaining brain of ageing WT mice with western blotting. Representative immunoblots for Sortilin, full-length and truncated TrkB, p75NTR and the internal loading control GAPDH (**A**). No differences in Sortilin were detected across the groups (*p* = 0.3886) (One-way ANOVA) (**B**). Full-length TrkB was also unchanged with age (*p* = 0.0808) (One-way ANOVA) (**C**). Truncated TrkB was significantly increased in mice aged 12 months relative to both 6 months (*p* = 0.0015) and 9 months (*p* = 0.0231) (One-way ANOVA) (**D**). Mice aged 14 months also had significantly increased truncated TrkB relative to 6 months (*p* = 0.0127) (**D**). No changes in p75NTR could be found across the groups (*p* = 0.0648) (One-way ANOVA) (**E**). All groups consisted of 6 mouse samples except the 6 months age group, which consisted of 5 samples. Data presented as mean ± SEM, *p < 0.05, **p < 0.01, ***p < 0.001, ****p < 0.0001

Like other brain regions, except the pre-frontal cortex, Sortilin was remarkably stable with age in the remaining brain (Fig. 7B). Full-length TrkB showed a steady increase with age, though none of the changes were significant (Fig. 7C). By contrast, the truncated isoform was increased in mice aged 12 months relative to both 9 and 6 months (Fig. 7D). There was also a finding of increased expression in mice aged 14 months relative to 6 months (Fig. 7D). These results demonstrate that truncated TrkB increases with age in WT mice while the full-length isoform remains unchanged. As for other brain regions, p75NTR was detectable in the remaining brain tissue (Fig. 7A). Although some age-related changes in the receptor were observed, none of them were significant (Fig. 7E). Taken together, p75NTR did not change with age in the remaining brain tissue of WT mice.

### Attenuation of p75NTR Signaling had no Effect on proBDNF Conversion in most Brain Regions

**Fig. 8:**
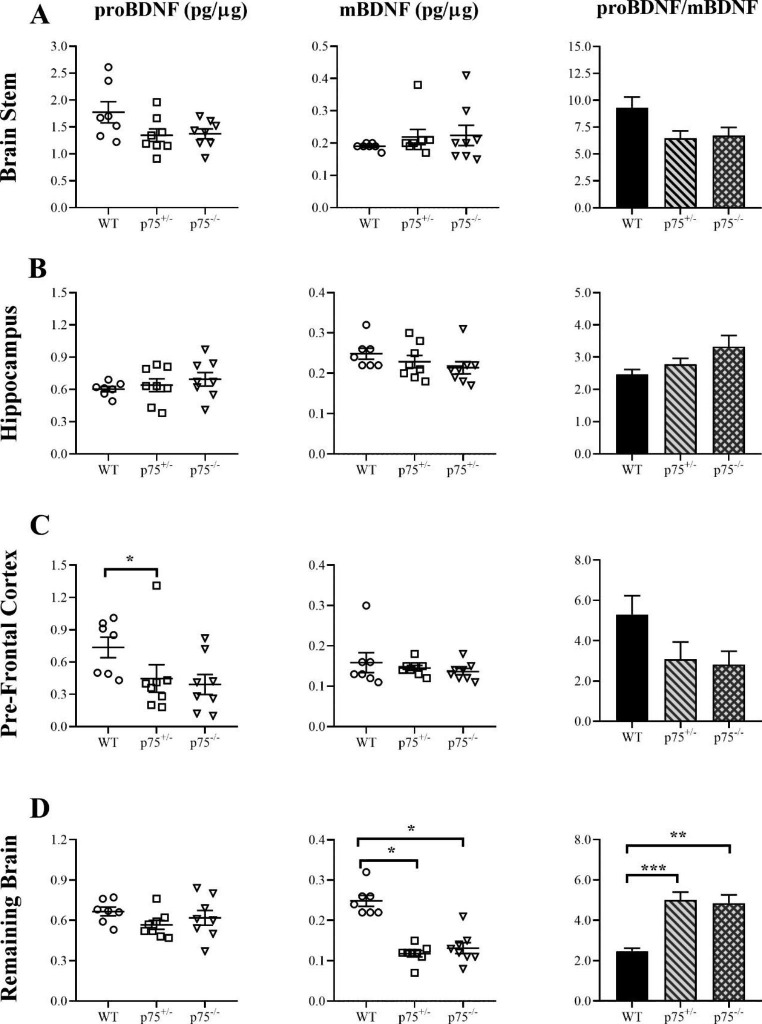
Investigating the involvement of p75NTR signaling on BDNF conversion using ELISA. No changes in proBDNF (*p* = 0.0763) (One-way ANOVA) or mBDNF (*p* = 0.4763) (Kruskal-Wallis) were detected across the genotypes in the brain stem (**A**). The pro/mature BDNF ratio also remained unchanged across the groups (*p* = 0.0486) (One-way ANOVA) (**A**). In the hippocampus, proBDNF (*p* = 0.5039) (One-way ANOVA) and mBDNF (*p* = 0.1023) (Kruskal-Wallis) also remained unchanged across groups, as did the pro/mature BDNF ratio (*p* = 0.0730) (One-way ANOVA) (**B**). There was a significant reduction in proBDNF for p75^+/−^ mice in the pre-frontal cortex compared to WT (*p* = 0.0291) (Kruskal-Wallis) (**C**). However, mBDNF remained unchanged (*p* = 0.6015) (Kruskal-Wallis) (**C**), as did the pro/mature BDNF ratio. In the remaining brain, proBDNF was unchanged (*p* = 0.2826) (One-way ANOVA) (**D**) whereas mBDNF was reduced in both p75^+/−^ and p75^−/−^ mice relative to WT (*p* = 0.0015) and (*p* = 0.0054) (Kruskal-Wallis) respectively (**D**). The pro/mature BDNF ratio was significantly increased in both p75^+/−^ and p75^−/−^ mice compared to WT (*p* = 0.0005) and (*p* = 0.0012) (One-way ANOVA) respectively (**D**). Each open shape represents an individual mouse sample. Data presented as mean ± SEM, *p < 0.05, **p < 0.01, ***p < 0.001, ****p < 0.0001

proBDNF and mBDNF were measured in p75^-/-^ mice to determine if p75NTR signaling influences proBDNF conversion. The previous analysis of p75NTR in this study suggested that the receptor does not change with age, except in the hippocampus. However, total protein levels do not reflect signaling, so this analysis could not determine if p75NTR signaling had changed. proBDNF mediates its neurotoxic effects through p75NTR, so an increase in its relative amount suggests that p75NTR signaling would also be increased (Marchetti et al. 2019). An attenuation of p75NTR signaling, therefore, might counter the effects of higher levels of proBDNF. To determine if p75NTR signaling influences the pro/mature BDNF ratio, BDNF isoforms were measured in p75^-/-^ mice. Heterozygous mice (p75^+/-^) were included to determine to what extent it influences the ratio as p75NTR signaling would be partially impaired in these mice. proBDNF and mBDNF remained unchanged in the brain stem and hippocampus across genotypes (Fig. 8A and B). Accordingly, the pro/mature BDNF ratio was also unchanged. A reduction in proBDNF was found in the pre-frontal cortex for p75^+/-^ mice compared to WT, but not p75^-/-^, while mBDNF remained stable across the genotypes (Fig. 8C). Although there were no changes in proBDNF in the whole brain, mBDNF was reduced for both p75^+/-^ and p75^-/-^ mice compared to WT (Fig. 8D). The decreased levels of mBDNF resulted in an increased pro/mature BDNF ratio for both genotypes relative to WT. Taken together, impairments in p75 signaling had no effect on BDNF conversion in most brain regions.

## Discussion

This study investigated changes in proBDNF relative to mBDNF in brain regions of young to middle-aged WT mice. proBDNF was increased relative to its mature isoform in several brain regions, except the hippocampus, suggesting that a neurotrophic imbalance occurs as early as middle age. However, the increasing ratio did not correspond with trends in each respective BDNF receptor or with the sorting receptor sortilin. Consequently, the hypothesis that proBDNF would increase in several brain regions was only partly supported. An unexpected finding was that BDNF isoforms in the hippocampus did not follow the same trendlines as other brain regions. Impairments in hippocampal neurogenesis, reduced CA1 dendritic spine density, and lower expression of plasticity-related genes occur in middle-aged WT mice (Bensalem et al. 2016; Furukawa et al. 2021; Weber et al. 2015). In addition, deficits in working memory have also been identified (Shoji and Miyakawa 2019), but changes in spatial navigation are less clear (Bensalem et al. 2016; Boyer et al. 2019; Furukawa et al. 2021; Long et al. 2009). Given the role of proBDNF in hippocampal dendritic spine loss (Yang et al. 2014), it was surprising to find that it remained stable relative to its mature isoform. A possible explanation for this unexpected result could be plasticity of the hippocampus due to its involvement in learning and memory. proBDNF is converted by intracellular and extracellular proteases during different stages of LTP in hippocampal neurons (Pang et al. 2016). Despite the deficits in synaptic plasticity in middle-aged mice, it may take some time for these impairments to manifest as increasing relative amounts of proBDNF. A difference between this study and others was that pro/mature BDNF was the main outcome measure and changes only measured up to middle-age. Most studies use individual isoforms as the outcome measures and continue the analysis into old age (Buhusi et al. 2017; Calabrese et al. 2013; Perovic et al. 2013). The ratio better reflects how proBDNF changes relative to its mature isoform over time.

The finding of a close relationship between the pro/mature BDNF ratio and brain age could be important for studies involving humans. The use of peripheral BDNF levels as a proxy of brain age has been a focus of research in humans. These studies measure associations between serum BDNF and age to determine if declining levels of the protein are reflective of brain age (Erickson et al. 2010; Naegelin et al. 2018; Puhlmann et al. 2021). So far, these studies have yielded conflicting results. Some show a negative correlation between serum BDNF and age while others show a positive correlation and yet others, more recently, show no correlation (Erickson et al. 2010; Naegelin et al. 2018; Puhlmann et al. 2021). These conflicting results could be because BDNF levels in the periphery do not reflect levels in the brain. BDNF is secreted by the adipose tissue that surrounds arteries into circulation, where it then binds to receptors on smooth muscle cells (Zierold et al. 2021). Consequently, excess adipose deposition could lead to higher levels of serum BDNF, which may mask reductions in the brain. However, another explanation is that individual BDNF isoforms do not reflect brain age as closely as the pro/mature BDNF ratio. This explanation was supported in this study, which showed a relationship between the pro/mature BDNF ratio and age in all brain regions except the brain stem. The relationship was especially strong in the pre-frontal cortex, which could explain the dendritic spine loss that occurs in this region. Dendritic spine loss in Brodmann area 46 of the dorsolateral pre-frontal cortex negatively correlates with age in humans without neuropathology (Boros et al. 2019). Given the role of proBDNF in mediating LTD, which leads to the shrinkage of dendritic spines (Garad et al. 2021), an age-related increase in its relative expression could explain their gradual loss. Future studies in humans and rodents could make a stronger connection between the pro/mature BDNF ratio and age-related dendritic spine loss.

The hypothesis was partly supported due to the lack of concordance between BDNF isoforms and their receptors. This study identified clear trends of increasing amounts of proBDNF relative to mBDNF in several brain regions of middle-aged mice. However, this increasing ratio did not correspond with trends in each respective BDNF receptor. Levels of proBDNF peak at post-natal day 24 in the rat hippocampus then decline to basal levels and remain stable until 2 months (Sun et al. 2021). The high levels of proBDNF correspond to high levels of p75NTR, the receptor that mediates its effects, which is when its activity is high (Yang et al. 2014). In this study, the increasing relative amounts of proBDNF did not correspond with trends in p75NTR. Moreover, sortilin, which functions as a co-receptor for proBDNF/p75NTR-mediated apoptosis (Nykjaer and Willnow 2012), was stable in most brain regions. Nevertheless, relative amounts of a receptor do not reflect its signaling activity, so it remains uncertain whether the higher relative levels of proBDNF corresponded with increased p75NTR signaling. Levels of mBDNF across age also didn’t correspond to changes in the receptor that mediates its neurotrophic effects, full-length TrkB. mBDNF increases the length, head-width, and density of dendritic spines in mature pyramidal neurons of the hippocampus through TrkB (Kellner et al. 2014). mBDNF remained remarkably stable in brain regions that it was measured in while changes in full-length TrkB were observed. However, total protein levels do not reflect receptor signaling, so it could not be established if mBDNF/TrkB signaling was reduced.

The mostly unchanged pro/mature BDNF ratio in p75 mutant mice suggests that p75NTR signaling had no effect on their relative levels. Although some changes were observed in p75 mutant mice, these were either inconsistent across brain regions or contrary to the hypothesis. proBDNF was reduced in the pre-frontal cortex of p75^+/-^ mice, but not p75^-/-^, suggesting that partial impairment of p75NTR signaling reduces proBDNF, but not complete impairment. In the remaining brain, mBDNF was reduced in p75^+/-^ and p75^-/-^ mice, and the pro/mature BDNF ratio increased, suggesting that p75NTR signaling maintains levels of the mature isoform. This last result suggested, paradoxically, that p75NTR signaling maintains the balance between pro and mature BDNF. The lack of changes in other brain regions, especially the hippocampus, indicated that p75NTR signaling did not influence BDNF isoforms in regions relevant to AD. Consequently, the hypothesis that impaired p75NTR signaling would restore the pro/mature BDNF ratio was not supported. This negative finding could be because the effector proteins of p75NTR are downstream from proBDNF and have no influence on proteases that convert the precursor. Future studies should investigate factors that influence the activity of proteases involved in BDNF maturation to determine why there is an imbalance in middle-aged mice.

A limitation of this study was the lack of aged mice to determine how the pro/mature BDNF ratio changes in old age. Most male wild-type mice (75%) live for 24 months in a pathogen-free, controlled environment while females live for 22 months (Turturro et al. 1999). Mice aged 10–14 months are representative of middle-aged humans (38–47 years), whereas mice aged 18–24 months represent old humans (56–69 years) (Flurkey et al. 2007). Therefore, the mice aged 14 months in this study, the oldest age category, were equivalent to humans aged in their late forties. Because no old mice were included, it is unknown how the pro/mature BDNF ratio changes in old age.

## Conclusion

This study has shown that proBDNF increases relative to mBDNF with normal age in several brain regions of WT mice. It could not be determined why the imbalance occurred, which remains an open question. The pro/mature BDNF ratio was also found to correlate with brain age more closely than individual isoforms in several brain regions. This finding could be important for studies in humans which aim to develop biomarkers of brain ageing.

## References

[CR1] Aarse J, Herlitze S, Manahan-Vaughan D (2016). The requirement of BDNF for hippocampal synaptic plasticity is experience-dependent. Hippocampus.

[CR2] Adler DH, Wisse LEM, Ittyerah R (2018). Characterizing the human hippocampus in aging and Alzheimer’s disease using a computational atlas derived from ex vivo MRI and histology. Proc Natl Acad Sci USA.

[CR3] Arvanitakis Z, Shah RC, Bennett DA (2019) Diagnosis and management of Dementia. Review’ JAMA 322 161589–1599. 10.1001/jama.2019.4782 10.1001/jama.2019.4782 PMC7462122 31638686

[CR4] Bensalem J, Servant L, Alfos S (2016). Dietary polyphenol supplementation prevents alterations of spatial navigation in middle-aged mice. Front Behav Neurosci.

[CR5] Boros BD, Greathouse KM, Gearing M (2019). Dendritic spine remodeling accompanies Alzheimer’s disease pathology and genetic susceptibility in cognitively normal aging. Neurobiol Aging.

[CR6] Boyer F, Jaouen F, Ibrahim EC (2019). Deficits in social behavior precede cognitive decline in middle-aged mice. Front Behav Neurosci.

[CR7] Braak H, Braak E (1995). Staging of Alzheimer’s disease-related neurofibrillary changes. Neurobiol Aging.

[CR8] Buhusi M, Etheredge C, Granholm AC (2017). Increased hippocampal proBDNF contributes to memory impairments in aged mice. Front Aging Neurosci.

[CR9] Calabrese F, Guidotti G, Racagni G (2013). Reduced neuroplasticity in aged rats: a role for the neurotrophin brain-derived neurotrophic factor. Neurobiol Aging.

[CR10] Chan CB, Ye K (2017). Sex differences in brain-derived neurotrophic factor signaling and functions. J Neurosci Res.

[CR11] Chen ZY, Ieraci A, Teng H (2005). Sortilin controls intracellular sorting of brain-derived neurotrophic factor to the regulated secretory pathway. J Neurosci.

[CR12] De Strooper B, Karran E (2016). The cellular phase of Alzheimer’s Disease. Cell.

[CR13] Dokter M, Busch R, Poser R (2015). Implications of p75NTR for dentate gyrus morphology and hippocampus-related behavior revisited. Brain Struct Funct.

[CR14] Erickson KI, Prakash RS, Voss MW (2010). Brain-derived neurotrophic factor is associated with age-related decline in hippocampal volume. J Neurosci.

[CR15] Flurkey K, Currer JM, Harrison DE (2007) Mouse models in ageing research. In: Fox JG et al (eds) The mouse in biomedical research, 2 edn. Elsevier, pp 637–672

[CR16] Frisen J, Verge VMK, Fried K (1993). Characterization of glial trkB receptors: Differential response to injury in the central and peripheral nervous systems. Proceed Natl Acad Sci USA.

[CR17] Furukawa T, Nikaido Y, Shimoyama S (2021). Impaired cognitive function and hippocampal changes following chronic diazepam treatment in middle-aged mice. Front Aging Neurosci.

[CR18] Garad M, Edelmann E, Lessmann V (2021). Long-term depression at hippocampal mossy fiber-CA3 synapses involves BDNF but is not mediated by p75NTR signaling. Sci Rep.

[CR19] Kellner Y, Godecke N, Dierkes T (2014). The BDNF effects on dendritic spines of mature hippocampal neurons depend on neuronal activity. Front Synaptic Neurosci.

[CR20] Lim Y, Zhong JH, Zhou XF (2015). Development of mature BDNF-specific sandwich ELISA. J Neurochem.

[CR21] Long LH, Liu RL, Wang F (2009). Age-related synaptic changes in the CA1 stratum radiatum and spatial learning impairment in rats. Clin Exp Pharmacol Physiol.

[CR22] Marchetti L, Bonsignore F, Gobbo F (2019). Fast-diffusing p75(NTR) monomers support apoptosis and growth cone collapse by neurotrophin ligands. Proc Natl Acad Sci USA.

[CR23] Martorana F, Gaglio D, Bianco MR (2018). Differentiation by nerve growth factor (NGF) involves mechanisms of crosstalk between energy homeostasis and mitochondrial remodeling. Cell Death Dis.

[CR24] Medcalf RL (2017). Fibrinolysis: from blood to the brain. J Thromb Haemost.

[CR25] Naegelin Y, Dingsdale H, Sauberli K et al (2018) Measuring and validating the levels of brain-derived neurotrophic factor in human serum. eNeuro. 5(2):1–9. 10.1523/eneuro.0419-17.2018 10.1523/ENEURO.0419-17.2018 PMC5898630 29662942

[CR26] Niculescu D, Michaelsen-Preusse K, Guner U (2018). A BDNF-mediated push-pull plasticity mechanism for synaptic clustering. Cell Rep.

[CR27] Nykjaer A, Willnow TE (2012). Sortilin: a receptor to regulate neuronal viability and function. Trends Neurosci.

[CR28] Obiang P, Maubert E, Bardou I (2011). Enriched housing reverses age-associated impairment of cognitive functions and tPA-dependent maturation of BDNF. Neurobiol Learn Mem.

[CR29] Pang PT, Nagappan G, Guo W (2016). Extracellular and intracellular cleavages of proBDNF required at two distinct stages of late-phase LTP. NPJ Sci Learn.

[CR30] Perovic M, Tesic V, Mladenovic Djordjevic A (2013). BDNF transcripts, proBDNF and proNGF, in the cortex and hippocampus throughout the life span of the rat. Age.

[CR31] Petkova-Tuffy A, Godecke N, Viotti J (2021). Neuroligin-1 mediates presynaptic maturation through brain-derived neurotrophic factor signaling. BMC Biol.

[CR32] Puhlmann LMC, Linz R, Valk SL (2021). Association between hippocampal structure and serum brain-derived neurotrophic factor (BDNF) in healthy adults: a registered report. NeuroImage.

[CR33] Rauti R, Cellot G, D’Andrea P (2020). BDNF impact on synaptic dynamics: extra or intracellular long-term release differently regulates cultured hippocampal synapses. Mol Brain.

[CR34] Schmitz TW, Spreng NR, Alzheimer’s Disease Neuroimaging Initiative (ADNI) (2016). Basal forebrain degeneration precedes and predicts the cortical spread of Alzheimer’s pathology. Nat Commun.

[CR35] Shoji H, Miyakawa T (2019). Age-related behavioral changes from young to old age in male mice of a C57BL/6J strain maintained under a genetic stability program. Neuropsychopharmacol Rep.

[CR37] Sun Y, Lim Y, Li F (2012). ProBDNF collapses neurite outgrowth of primary neurons by activating RhoA. PLoS ONE.

[CR36] Sun W, Cheng H, Yang Y (2021). Requirements of postnatal proBDNF in the hippocampus for spatial memory consolidation and neural function. Front Cell Dev Biol.

[CR38] Tessarollo L, Yanpallewar S (2022). TrkB truncated isoform receptors as transducers and determinants of BDNF functions. Front Neurosci.

[CR39] Timmers T, Ossenkoppele R, Wolters EE (2019). Associations between quantitative [(18)F]flortaucipir tau PET and atrophy across the Alzheimer’s disease spectrum. Alzheimers Res Ther.

[CR40] Turturro A, Witt WW, Lewis S (1999). Growth curves and survival characteristics of the animals used in the biomarkers of aging program. J Gerontol Biol Sci.

[CR41] von Bohlen O, Minichiello L, Unsicker K (2003). Haploinsufficiency in trkB and/or trkC neurotrophin receptors causes structural alterations in the aged hippocampus and amygdala. Eur J Neurosci.

[CR42] von Schack D, Casademunt E, Schweigreiter R (2001). Complete ablation of the neurotrophin receptor p75NTR causes defects both in the nervous and the vascular system. Nat Neurosci.

[CR43] Weber M, Wu T, Hanson JE et al (2015) Cognitive deficits, changes in synaptic function, and brain pathology in a mouse model of normal aging. eNeuro. 2(5):1–26. 10.1523/ENEURO.0047-15.2015 10.1523/ENEURO.0047-15.2015 PMC4606159 26473169

[CR44] Yang J, Harte-Hargrove LC, Siao CJ (2014). proBDNF negatively regulates neuronal remodeling, synaptic transmission, and synaptic plasticity in hippocampus. Cell Rep.

[CR45] Zhou L, Xiong J, Lim Y (2013). Upregulation of blood proBDNF and its receptors in major depression. J Affect Disord.

[CR46] Zierold S, Buschmann K, Gachkar S (2021). Brain-derived neurotrophic factor expression and signaling in different perivascular adipose tissue depots of patients with coronary artery disease. J Am Heart Assoc.

